# Implantable cardioverter-defibrillators might not be necessary in all patients with idiopathic ventricular fibrillation

**DOI:** 10.1007/s12471-024-01898-0

**Published:** 2024-09-06

**Authors:** Alwin B. P. Noordman, Alexander H. Maass

**Affiliations:** University Medical Centre Groningen, Department of Cardiology, University of Groningen, Groningen, The Netherlands

Dear editor,

Verheul et al. present their important data from a Dutch registry of patients with idiopathic ventricular fibrillation (iVF) [1]. They underline the importance of comprehensive diagnostic testing to find a specific diagnosis. An underappreciated topic is prediction of future arrhythmic events as 74% of their patients do not experience appropriate ICD therapy. These young patients are prone to complications due to risk of electrode failure and need for multiple future interventions.

We previously demonstrated that secondary prevention patients with an unclear cause of ventricular arrhythmia received significantly less appropriate ICD therapy compared to those with a clear cause (14% vs. 41%). Considering that the rate of first appropriate therapy was highest in the first two years, [2] patients who do not receive appropriate therapy within the lifetime of their first ICD may not derive sufficient benefit to warrant ICD replacement.

We hope that the Dutch iVF registry can provide predictors for future arrhythmic events to optimally advise patients in the difficult decision-making process surrounding ICD replacement [3]. If we can identify patients with low risk of recurrence, ICD implantation may even be omitted altogether.
